# Stakeholders’ views on the most and least helpful aspects of the ICH E6 GCP guideline and their aspirations for the revision of ICH E6(R2)

**DOI:** 10.1016/j.conctc.2022.100983

**Published:** 2022-08-17

**Authors:** Carrie Dombeck, Teresa Swezey, Annemarie Forrest, Pamela Tenaerts, Amy Corneli

**Affiliations:** a Clinical Trials Transformation Initiative, Duke University, Durham, NC, USA; b Department of Population Health Sciences, Duke University School of Medicine, Durham, NC, USA; c Duke Clinical Research Institute, Duke University School of Medicine, Durham, NC, USA

**Keywords:** Good clinical practice, Research design, Clinical trials, ICH E6

## Abstract

**Background:**

The International Council for Harmonisation of Technical Requirements for Pharmaceuticals for Human Use (ICH) has published the ICH E6(R2) Good Clinical Practice (GCP) guideline, which provides standards for the design, conduct, documentation, and reporting of clinical trials. Revision to E6(R2) is currently underway, aiming to adapt the guidance to the current regulatory environment. The Clinical Trials Transformation Initiative (CTTI) interviewed stakeholders, gathering their experiences implementing ICH E6 GCP and suggestions for revising the guidance.

**Methods:**

We conducted a qualitative descriptive study using in-depth interviews. Participants were purposefully selected to ensure diversity in geography, research role, and type of institution. Participants reflected on their aspirations for the ICH E6 GCP revision and described sections of the guidance that they found most and least helpful. Narratives were analyzed using applied thematic analysis.

**Results:**

Many participants found ICH E6 GCP generally clear and helpful. They appreciated that the guidance is globally accepted and serves as a common standard for research worldwide. Participants also noted opportunities for improvement, suggesting that the revised guidance should incorporate flexibility, simplify requirements, and accommodate advances in research conduct. They highlighted areas where language should be updated and concepts clarified and expressed a desire for transparency and inclusiveness in the revision process.

**Conclusion:**

Our findings show that many participants view the ICH E6(R2) guidance as helpful overall, although substantial room for improvement remains. We have provided the full report of these findings to ICH in hopes that it will be useful as the E6 GCP guideline is revised.

## 1 Introduction

The International Council for Harmonisation of Technical Requirements for Pharmaceuticals for Human Use (ICH) aims to achieve greater harmonization worldwide for developing safe, effective, and high-quality medicines [1]. ICH has published numerous guidelines to facilitate its mission, including the ICH E6 Good Clinical Practice (GCP) guideline [2], which provides standards for the design, conduct, documentation, and reporting of clinical trials. The guideline is intended for clinical trials with data collected for regulatory submission but states that the general principles “may also be applied to other clinical investigations.” ICH E6 GCP was first released in 1996 as E6(R1), and an integrated addendum to the guidance, E6(R2), was issued in 2016. This addendum was intended to address evolutions in technology and clinical trial practices occurring since the (R1) release and to encourage greater efficiency in clinical trials.

Several critiques of ICH E6 GCP have been identified, including lack of flexibility [3,4], confusion around application of the guidelines to non-regulatory trials [3], inconsistencies between the original guidance and the (R2) addendum [3], challenges with implementing the guidance in low- and middle-income countries [[4], [5], [6]], and lack of transparency and stakeholder inclusiveness [3,7]. Updates to the E6(R2) guidance aimed at acknowledging and addressing many of these criticisms are currently underway [8]. ICH describes that the goal of this revision is to better adapt to the current regulatory environment by “addressing the application of GCP principles to the increasingly diverse trial types” and data sources being utilized and by providing “flexibility … to facilitate the use of technological innovations in clinical trials” [9]. Where appropriate, the revision will preserve concepts and guidance from the previous version but will include modifications aimed at providing guidance for a diversity of approaches to clinical trials, as well as address gaps and inconsistencies [9].

The Clinical Trials Transformation Initiative (CTTI) conducted a multi-method project aimed at providing ICH with recommendations on ICH E6 GCP from a diverse group of stakeholders representing academic, pharmaceutical, and other institutions engaged in the conduct of clinical trials. CTTI is a public-private partnership between Duke University and the United States (U.S.) Food and Drug Administration that develops and drives the adoption of practices to increase the quality and efficiency of clinical trials. CTTI independently conducted a 3-phase project consisting of (1) a global online survey, (2) qualitative, in-depth telephone interviews, and (3) an open comment platform [10]. The purpose of these activities was to gather stakeholders’ perceptions of areas in ICH E6 GCP that are in greatest need of revision, their experiences with implementing ICH E6 GCP, and their suggestions for revising the guidance. This paper describes the findings from the in-depth qualitative interviews; the results from the other phases of the project are available elsewhere [11,12].

## 2 Material and methods

### 2.1 Study design

We conducted a qualitative descriptive study [13,14] using in-depth interviews (IDIs).

### 2.2 Participant eligibility and selection

Stakeholders were eligible to participate in the IDIs if they completed the online survey and self-reported that they 1) regularly reference ICH E6 GCP to implement their research, 2) conduct research for registrational purposes, 3) were willing to have the information they provided in the IDI be linked to their name and organization, and 4) were interested in participating in a follow-up IDI and share their experiences with implementing ICH E6 GCP and suggestions for its improvement. Among these eligible stakeholders, we purposefully selected and invited participants for the IDIs to ensure that the sample was diverse in geographic location of employment, countries where participants conducted research, role in research (e.g., investigator, clinical operations personnel, quality assurance personnel), and type of institution (e.g., university/academic center, pharmaceutical company, contract research organization [CRO]).

### 2.3 Data collection

A trained qualitative interviewer conducted the IDIs in English and on the telephone from September 15 to November 29, 2019. Reflecting on the 2016 ICH E6(R2) addendum, participants were asked to describe (1) their overall hopes for what the revision to ICH E6 GCP will achieve, (2) sections of ICH E6 GCP that they have found most helpful and why, including examples of how they have applied the guidance, (3) sections of ICH E6 GCP that have been least helpful to them and why, including examples of difficulties encountered in applying the guidance, and (4) suggestions for how ICH E6 GCP guidance could be improved and how this would subsequently improve trial conduct. Although participants were not asked to reflect specifically on their survey responses, when requested by the participant, or if the participant needed encouragement to respond to the interview questions, the interviewer reminded participants about the areas of ICH E6 GCP they previously indicated in the survey were in need of revision.

### 2.4 Data analysis

We used descriptive statistics to summarize the demographic data and applied thematic analysis [15] to analyze participants’ IDI narratives. All interviews were transcribed verbatim following a transcription protocol [16]. We used NVivo 12 [17], a qualitative data analysis software program, to organize the data and apply codes to the transcripts. First, two analysts developed and independently applied structural codes based upon the three primary research questions: 1) aspirations for the revision of ICH E6 GCP, 2) helpful sections, and 3) unhelpful sections and suggested revisions. These structural codes were further segmented into sub-codes for 1) each of the eight sections of ICH E6 GCP, and 2) general comments about each of the research questions. Inter-coder agreement was assessed on five interviews (22%), discrepancies in code application were resolved through discussion, and where necessary, structural coding was revised accordingly.

Next, the analysts identified content codes within each structural code that reflected participants' aspirations, experiences, and suggestions for updating ICH E6(R2). The sub-sections of ICH E6 GCP were used to categorize these content codes while general comments were coded separately. Analysts also identified overarching themes, content codes that emerged across numerous sub-sections of ICH E6 GCP. Code frequency was reviewed to identify participants’ main experiences and suggestions. While several important themes were identified based on frequency, many valuable experiences and suggestions were shared by only a small number of individual participants. All IDI participants were well-positioned to provide helpful information based on their unique experiences and insights; therefore, we included most participant comments in the final CTTI report [18] and highlighted the most salient findings here.

### 2.5 Ethics

The Duke University Health System Institutional Review Board (IRB) determined that the research was exempt from further IRB review.

## 3 Results

### 3.1 Participant characteristics

Of the 327 individuals who completed the survey, 75 agreed to be contacted for IDIs, and 23 participated in the IDIs. Participants were employed in 10 different countries (Table 1): Argentina, Australia, Belgium, Canada, France, Germany, Ireland, Switzerland, United Kingdom, and the US. Similar to the survey population [11], most interview participants were from Europe and North America. Participants conducted research in 124 countries worldwide (Supplemental Appendix A). Participants also held various research roles, including principal investigator, co-investigator, and clinical operations, quality assurance, and regulatory affairs personnel, and represented different types of institutions, such as academic research centers, pharmaceutical companies, CROs, and non-governmental organizations (Table 1).

**Table 1:** Participants’ engagement in research (n = 23).

**Geographic location of employment**	**n (%)**
East Asia and Pacific	2 (8.7)
Australia	2 (8.7)
Europe and Central Asia	13 (56.5)
Belgium	2 (8.7)
France	2 (8.7)
Germany	4 (17.4)
Ireland	1 (4.3)
Switzerland	2 (8.7)
United Kingdom	2 (8.7)
Latin America and Caribbean	1 (4.3)
Argentina	1 (4.3)
North America	7 (30.4)
Canada	3 (13.0)
United States of America	4 (17.4)

**Type of institution**	**n (%)**

University/academic research center affiliated with a hospital/medical center	8 (34.8)
Contract research organization (commercial/for profit)	5 (21.7)
Pharmaceutical company or biotechnology company	4 (17.4)
Non-governmental organization or not-for-profit organization	3 (13.0)
Hospital/medical center not affiliated with a university/academic research center	2 (8.7)
Trade/professional organization	1 (4.3)

**Main research role**	**n (%)**

Clinical operations personnel	7 (30.4)
Principal investigator, co-investigator, sub-investigator, site investigator	5 (21.7)
Quality assurance/quality control personnel	5 (21.7)
Regulatory affairs personnel	4 (17.4)
Clinical research associate/research coordinator/study nurse	1 (4.3)
Data analyst	1 (4.3)

### 3.2 Helpful aspects of the ICH E6 GCP guidance

Many participants stated that ICH E6 GCP is helpful, generally clear, and useful for training. Participants described that ICH E6 GCP represents the only globally accepted guidance, serving as a common standard for research worldwide, and they stated that it is particularly useful for establishing a research framework in countries with under-developed legal or regulatory requirements for trials, or where variation in regulations exists between countries. They explained that having this common framework is helpful for ensuring that globally generated trial data complies with requirements for product registration and is meaningful for marketing organization applications. Participants also emphasized that information on human subjects protections sets an effective standard for protecting participants’ rights, safety, and welfare. Participants noted that they found the sections on GCP principles, investigator and sponsor responsibilities, and essential documents to be particularly helpful, stating that these sections contain especially clear guidance, as well as templates and checklists of essential elements. Within the sponsor section, participants said they particularly appreciated the guidance on quality management using a risk-based approach, stating that this has been useful to their work, and some found the shift to risk-based monitoring established in the (R2) revision to be clear and helpful (see Table 2, Section 1 for participant quotes).

**Table 2:** Participant quotes.

Topic	Participant quote
**Section 1: Helpful aspects of ICH E6 GCP**	

Globally accepted guidance	*Because of the work that I've undertaken, it's always been with CROs. The expectation has always been the data generated in these countries would be GCP-compliant, and that's necessary in order to support product registration in the EU at the very least. And so, for that reason, when we've been working with the sponsors, obviously, they've been wanting to recruit patients in these countries, but they've also been wanting to ensure that patients provide data that's meaningful to their ultimate marketing organization application. So, for that reason, we would ultimately have to follow ICH anyway given the fact that we do have to perform applications to these countries, and often the regulatory requirements and the ethics requirements in these countries are quite sketchy, at best.* —United Kingdom, CRO, regulatory affairs

Section 2: GCP principles	*… for all of our clinical work, the 13 principles are pretty important. It doesn't matter whether it's for a drug application or a device application. The principles, in general, are very good and a north star for clinical research.* —Canada, university/academic, clinical operations

Section 4: Investigator responsibilities	*… the informed consent form requirements laid out in ICH E6 are particularly useful. They provide a template, in essence, for how the documents should be written, and they make it easier for us to prepare a standard document that can be used across regions, which is a huge advantage, particularly when you're working in countries or regions where requirements aren't completely outlined. … And in countries where requirements aren't available, even those outside of ICH regions, the ICH template really becomes a gold standard there to ensure that you're conducting the research in a manner that would permit that data to be acceptable for use outside of that particular country …* —United Kingdom, CRO, regulatory affairs

Section 5: Sponsor responsibilities	*What else have I found to be helpful? I would say again the risk-based quality systems for sponsor … But even to see from the point of view of sponsor, to be able to say well okay, where are the possible risks in this trial that are like in the processes, etc. … I think looking at those up front before you even start means that we take on trials that are more suitable for, say, where we are and our population, etc., rather than taking on things and then realizing no, we've wasted that time on something that's not doable here. … It forces you to look at what are the possible risks and then to mitigate them before you start.* —Ireland, university/academic, regulatory affairs

Section 8: Essential documents	*No. 1 is section eight, which is essential documents. I use that all the time to make sure that the investigator site binder, that documents are collected at the right time, that our processes are defined based on when we collect those documents, and how they impact the rest of the study. So, that's a really important part of what I look at.* —Canada, CRO, clinical operations

**Section 2: Aspirations for the ICH E6 GCP revision**	

Incorporate flexibility	*And, obviously, also tailoring for different types of research. Sometimes, a different aspect of the ICH GCP doesn't relate to all types of research. And, it would be good if that could be part of the revision itself, [outlining what] sometimes is applicable and [what] sometimes is not.* —Australia, NGO or not-for-profit, clinical operations

*I am coming from an academic organization … E6 gets applied to our clinical research whether it's for a drug application or drug approval or not. Some of the type of work we do … [has] nothing to do with an approval of the drug … So, all GCP in general—the concept or the spirit definitely applies—and we implement that. But, sometimes the implementation, or the expectations of the reviewers, that are the key factors that come in [with GCP] are still expected to dot some i's and cross t's where it's not reasonable to do because of the type of study we have.* —Canada, university/academic, clinical operations

Simplify the guidance	*… I think this whole thing needs to be seriously simplified because I believe that the level of bureaucracy has hit such a point that even if people are quite intensively trained, it's so huge that even [when] willing to comply, people make mistakes because it becomes too cumbersome. Then, with all the legislation on top that [researchers] need to comply with, it's just becoming too much. And, people are so much focused on the documentation that they actually forget about the real protection.* —Belgium, NGO or not-for-profit, regulatory affairs

*What I'm going to hope is that beside keeping the overall intention, like protecting patients and ensuring data integrity, the guideline [will] move away … from a checklist exercise and a tool [that is] very much misused for audit and inspection to a document or series of documents [that] incorporates new technologies and new ways of working, but also enables investigators, academics, ethics committees, and sponsor a successful partnership with the outcome that we get … drugs to the patient faster, but that these drugs are safe.* —Germany, pharmaceutical or biotech, quality assurance

Accommodate advances in research conduct	*… since the guideline was first produced, there's been a tremendous advance in the way clinical trials are conducted now. Again, a lot of the approach within the guideline seems to be very much based on the traditional way of managing trials with paper, whereas we're obviously moving to a much more electronic environment now. Huge differences. And, I think while respecting the principles, there needs to be quite a change in approach to accommodate the new technology.* —United Kingdom, trade/professional organization, regulatory affairs

*My expectation of ICH E6 is whatever they're going to write … it's really taking into consideration how clinical trials are conducted today, but also, how they will look in the future … I think that's very tricky, especially if you take into consideration the long development process for ICH guidelines. The moment the guidelines are going to be finalized, they're already more or less outdated. Therefore, on one hand, you need to have a general document where the wording is high-level and can also be read for future technology, but on the other hand, the guidelines should give you enough detail that you know what you have to do …* —Germany, pharmaceutical or biotech, quality assurance

Update and clarify language	*I hope that actually the whole text will be revised and not only another addendum will be written…the addendum we now have some inconsistencies in terminologies, and we need to have this explanation in the introduction that if there is a conflict, then this version takes priority or something like this. I think this as GCP requires consistency throughout the document of a clinical trial. I think the guideline first becomes a stencil, so I have hope for a revision which actually goes through the full text and makes it more consistent.* —Germany, CRO, quality assurance

*… there is no mention of study coordinator in the GCP … study coordinators are critical to clinical research, and they carry a critical role for the success of the studies. I would like to see at least the study coordinator mentioned. … recognizing that importance in a study allows for study coordinators to feel empowered to do a better job and recognized. I think without them our research would not be possible.* —Argentina, CRO, clinical operations

Be transparent and inclusive	*… this is a major shortcoming in the previous ICH guidelines that patients and communities are not even mentioned as stakeholders in research. They are regarding investigators, sponsor, ethics committee, regulations, regulatory. But, I think in 2019, we already know very well how important it is to engage with communities and with patients to make research ethics pertinent, etc. So, it's pretty strange that they are not mentioned in the guidelines, as they should be, as stakeholders.* —Belgium, university/academic, CRA/research coordinator

*… one expectation would be, for the renovation, to have an explanation, elaboration document on the side that provides more explanation on how ICH arrived at this particular recommendation, and the rationale for it because it would make it easier for the people to understand, and then also to consciously deviate from it because they can then say, “Okay, look, it was implemented based on this background and this rationale, but in our situation, it's different, so we need to deviate,” or I can say “It's exactly that and it makes good sense.”* —Switzerland, university/academic, investigator

**Section 3: Unhelpful aspects of ICH E6 GCP**	

Scope of guidance	*It's really the scope of the ICH GCP because there is a lot of confusion out there. Should it only be for clinical trials with new medicines? Should it be with clinical trials with medicine and vaccine? Yeah, everybody agrees. But when it comes to the diagnostic research, I heard people saying, “Oh, no. The GCP are not applicable because it's not the medicine.” “Yeah, but you are testing the new test. So, it's important.” The other side, I have seen ethics committee referring to the GCP for social behavioral studies, which is not really the case. But, the principle can be applicable there because principles of informed consent or ethics review are checking the quality of data as easily applicable there. So now, it's a little bit vague. It is easy for clinical trials, but it can also be used for another kind of research. Perhaps, there should be more clarity on when it is really mandatory and when it is considered as an inspiration.* —Belgium, university/academic, CRA/research coordinator

Section 3: IRB/IEC	*One is not so helpful. I think it could be improved. … So, I think 3.2.1 where it says the IRB/IEC should consist of a reasonable number of members who collectively have the qualifications and experience to review and evaluate the science and medical aspects and ethics of the proposed trial. I think – finding these capabilities and resources to maintain IRB remains a challenge in worldwide locations with low study density. And there's dichotomy I think in many developing countries, depending on how ethics committees are structured. … They may be deficient in the number of members or the experience that they bring versus commercial IRBs that have a lot of volume. … And the way the training, qualification, and experience to review and evaluate the science doesn't really explain how much – how thorough this training should be. … the guideline may be more specific about the type of training and the type of training records to make sure we can have a robust training plan for the people who are doing the reviews and also mention the opportunity for the IRB to collaborate cooperatively and have remote members. To make it more possible to bring experienced qualifications and ethics and medical science knowledge to IRBs that maybe don't have the volume of protocols coming through their institutions. … From what I see in the countries where I work.* —Argentina, CRO, clinical operations

Section 4: Investigator responsibilities	*The other section that's challenging is looking at the investigator. So, the poor investigator, as we know, is responsible for almost everything. … If they're supervising anybody, it has to be cleared. But the other bit is if the investigator retains the services of any individual or party, that they have to basically ensure this individual is qualified. So, does that mean – and again, there have been various kind of discussions about interpretation – so, if an investigator is using, say, an outside body to carry out a test like an X-ray, an MRI, a lab, whatever – it almost implies that the investigator has to go audit that lab. And yet, they really don't have that skill or knowledge to be able to do that. So, I think that that's quite a big ask that if the investigator … Retains the services of any individual – to me, that's more like the sponsor. The sponsor is setting up the study, so why is it the investigator's responsibility? I think that can be looked at.* —Ireland, university/academic, regulatory affairs

Section 5: Sponsor responsibilities	*So, in [Section] 5.0.2, risk identification … I have not seen an industry-wide standardized approach to this because every sponsor decided on their own how to phrase that out. This is also what we did, and I don't know if our process would meet the expectations of the people who have written ICH …. We are missing now the thorough inspection on the new process if that would meet the expectations of the inspectorate …. That's the danger that I fear for the next revision. Something is implemented in best intent, but the uptake, really the proper setup to meet the expectations that are issued, is something that I'm a bit struggling around.*—Germany, pharmaceutical or biotech, clinical operations

Section 8: Essential documents	*… the whole issue [is] not about data capture and record capturing and TMF, [but] more about the archiving of TMFs. And, in some hospitals, there is no archive. This is then acceptable if the documentations archives are 100s of kilometers away in the capital where there is the climate control archive facility. [But] then it means that the investigator doesn't have easy access to the documentation in case there is some later AE or something where he needs to go back. So, I think that's something very, very difficult. Yes, we can organize an archive somewhere in the capital, but is that really still in the spirit why you need to archive at the site or close to the site, the site records?* —Switzerland, NGO or not-for-profit, data analyst

AE, adverse event; CRA, clinical research associate; CRO, contract research organization; GCP, Good Clinical Practice; EU, European Union; ICH, International Council for Harmonisation of Technical Requirements for Pharmaceuticals for Human Use; IEC, independent ethics committee; IRB, institutional review board; TMF, trial master file.

### 3.3 Aspirations for revision of the ICH E6 GCP guidance

Five themes emerged from participants' narratives on their aspirations for the revision: incorporate flexibility, simplify the guidance, accommodate advances in research conduct, update and clarify language, and be transparent and inclusive (see Table 2, Section 2 for participant quotes about aspirations). Many participants' narratives about unhelpful sections of the guidance also reflected these themes; thus, participants’ comments about suggested revisions for sections considered unhelpful are discussed here in conjunction with their aspirations for what revisions to the guidance should achieve (Table 2, Section 3 lists participant quotes on unhelpful aspects of ICH E6 GCP).

#### 3.3.1 Incorporate flexibility

Participants expressed a desire for flexibility in applying ICH E6 GCP. Many participants described uncertainty about whether or how the guidelines are intended to accommodate non-drug trials and other types of trials not intended for regulatory submission and discussed that globally, the guidelines are being strictly applied to many different types of research, including studies for which they may not be appropriate. As examples, participants mentioned non-Investigational Medicinal Product (IMP) trials for standard of care, post-marketing trials, post-authorization safety trials, pragmatic trials, and procedural studies. Because of this, participants described that the ICH E6 GCP revision should 1) be very specific about the types of research for which the full gamut of ICH E6 GCP is a requirement, 2) clarify when use of the full ICH E6 GCP is optional and therefore which components may be selected as appropriate for the needs of a particular study, and 3) provide a framework for adapting the GCP guidance to other types of research by identifying the minimum requirements necessary for different types of trials and setting quality standards that encompass non-interventional and non-drug studies.

Participants also described that as ICH E6 GCP is used globally, it would be helpful to acknowledge in the update that flexibility may be required when working in low- and middle-income countries. For example, they stated that it may be difficult to implement the full ICH E6 GCP in remote or under-resourced areas or in emergency settings. Likewise, participants explained that certain aspects of GCP may need to be adapted to accommodate the needs of vulnerable populations, such as informed consent with orphans or indigenous communities. An additional challenge to implementing GCP within these contexts includes a dearth of properly qualified and trained individuals who may be needed to compose an IRB in under-resourced countries or countries where few studies are conducted. To address this issue, participants suggested expanding the IRB and independent ethics committee (IEC) guidance to allow for collaborative IRB reviews, teleconferenced IRB meetings, and flexibility in training requirements for IRB members.

#### 3.3.2 Simplify the guidance

Participants described a desire for the updated guidance to be more user-friendly, including simplifying requirements for GCP refresher training and eliminating duplicative trainings required by sponsors. Participants commented that the complexity of the guidelines can serve as a disincentive for investigators and that the burden of trial complexity is viewed as particularly high by potential investigators, small single-site trials, and investigator-initiated studies. In their discussions of both aspirations for revision and unhelpful aspects of the guidance, participants emphasized that ICH E6 GCP should no longer be viewed as a highly prescriptive “checklist” that must be applied to all studies, which encourages it being used as a policing tool for audits and inspections, and viewed instead as a document based on the “spirit of GCP” that elucidates the organizing principles for research. Thus, participants suggested that an introductory preamble to the guidance, clearly stating that the intent is not prescriptive and reminding end users of the fundamental purposes of research and GCP—improving patient outcomes while protecting research participants and ensuring data integrity—would be helpful for arriving at a common understanding across users. Participants also requested that ICH E6 GCP provide templates, examples, scenarios, and best practices throughout its sections; provide additional direction on how to make protocols simpler and more feasible; and provide training materials focused on implementing the guideline.

#### 3.3.3 Accommodate advances in research conduct

Participants discussed that the update should accommodate changes in research conduct and technological innovations that have emerged since the guidelines were created. For example, participants suggested that the guidance should address different types of informed consent (e.g., delayed consent, waiver of consent, opt-out consent, e-consent) and different types of trials (e.g., multi-modality trials, such as those that incorporate both drugs and devices). Participants also requested guidance for working within new research frameworks enabled by advances in technology, such as paperless trials and remote data collection. For example, they expressed confusion about how to adapt ICH E6 GCP guidelines on investigator oversight, monitoring, and record-keeping to these new circumstances. Participants further expressed concern that inspections are not yet being conducted in accordance with E6(R2) but are still based on the 1996 criteria, leading to questions about whether sponsors have interpreted the (R2) guidance correctly. Participants felt ICH E6 GCP should also be updated to account for new or substantially altered study roles and responsibilities since the guidance was created (e.g., monitor, sponsor liaison, study coordinator) and should specifically include patients and community representatives as stakeholders. Participants further suggested that revisions be written at a sufficiently high level to ensure continued applicability in the future, given that technologies and processes continue to evolve rapidly.

#### 3.3.4 Update and clarify language

Participants pointed out the need to update and clarify terms or provide additional guidance within several sections of ICH E6 GCP. For example, within the section on GCP principles, participants suggested clarifying the meaning of retention requirements for the length of storage and the retention of different types of media. In the IRB/10.13039/100009228IEC section, they proposed additional guidance focusing on oversight of ethics committees, allocation of responsibility for monitoring site compliance between IRBs and sponsors, challenges related to composing and training IRBs in countries with low study density, and standards for conducting clinical research in public health emergencies. Participants additionally requested clarification of central terms and concepts, such as quality management using a risk-based approach and quality tolerance limits.

Participants noted that allocation of both investigator and sponsor responsibilities should be more clearly specified in the update, perhaps by adding individual subsections to their respective chapters. Some expansion of the investigator guidance was requested: for example, participants suggested updating the section on adequate resources to incorporate more flexibility in staff member roles and address the consequences of having inadequate resources. Lack of clarity was also perceived in the sponsor guidance, where participants described that individual sponsors variously interpret ICH E6 GCP, leading to over-resourcing both low- and high-impact risks to ensure GCP compliance and leading sponsors to implement increasingly complex quality control, quality management, and documentation requirements. Participants further requested updates to data privacy and record-keeping guidelines. Within the essential documents section, participants noted challenges related to guidance about the trial master file (TMF), including the challenge of creating and archiving a TMF in under-resourced countries, and requested further definitions and clarification of certain requirements. Participants additionally pointed out inconsistencies in terminology, noted where definitions in the ICH E6 GCP do not match definitions in other commonly accepted documents (e.g., “trial” vs. “study”), and requested that the E6 guidance be more fully integrated with other GCP “E” documents.

#### 3.3.5 Be transparent and inclusive

Participants emphasized a desire for transparency and inclusion in the revision process, stressing that it is important to include a wide variety of stakeholders, representing perspectives across the gamut of trials, to create guidance that is operationally feasible. As such, participants said patients and their communities should be included in the process of updating ICH GCP and stressed the importance of having sufficient numbers of stakeholders from each geographic region included. Participants also requested transparency surrounding creation of the revision, including in the process that will be followed, the rationale behind decisions, the stakeholders who are involved and how they were selected, the process for soliciting feedback throughout the revision, and what is done with any feedback received.

## 4 Discussion

Our findings show that while there is substantial room for improvement, many stakeholders view the GCP guidance in ICH E6(R2) as helpful overall. The global nature of the guidance provides a standard research framework that can be applied when working in countries with limited or varying research guidelines to ensure trial participant protections. IDI participants felt that ICH E6(R2) GCP clearly lays out how to establish an evidence base to ensure that trial data comply with regulatory requirements for product approval and helpfully delineates investigator and sponsor responsibilities and oversight. In particular, the transition in E6(R2) to risk-based monitoring was appreciated by participants.

However, participants also identified several ways in which the guidance can be further improved. Many of these points elaborate on previous criticisms of ICH E6 GCP [3] and reflect issues which are acknowledged in the pending E6(R2) revision [8]. Participants’ aspirations for the ICH E6 GCP revision include incorporating more details about the types of research for which full GCP is recommended and supplying the minimum standards that should be met for trials that do not fit those criteria, including non-interventional and non-drug studies in which researchers currently struggle to apply GCP. Updates designed to acknowledge current and future changes in technology, trial types, informed consent, study roles and responsibilities, data collection methods, and data sources since the original guidance was created would also render the revision more applicable to real-world settings. Participants stated that the revision should strive for better integration with other GCP “E” documents and aim to further increase usability by decreasing complexity, with an overall goal of moving toward the spirit of guiding principles of GCP and away from a strict checklist approach.

Participants further highlighted several inconsistencies and points of confusion around central concepts and terms that ICH should seek to clarify in the next iteration of the guidance, including principles around data privacy and retention requirements, investigators’ responsibility for adequate resources, best practices for monitoring, and quality management using a risk-based approach. The guidance should also encourage regulatory authorities to base inspections on the most recent version of GCP. Finally, participants discussed their concerns, echoed in the literature [3,7], that the revision of ICH E6 GCP should be transparent in its operations and encompassing of a wide variety of stakeholders, including patients, geographically diverse communities, and representatives across the trial spectrum, to achieve inclusive and operationally feasible guidance.

Independent of these research findings, steps have already been taken to address some of these issues. As part of the planned (R3) revision, ICH has committed [9] to addressing advances in technology and trial design and incorporating considerations for non-traditional trials while aligning with the principles and objectives of the existing guidance and addressing identified gaps and inconsistencies. The end result will take into account the diversity of clinical trials and will highlight both that “achieving GCP principles and objectives can be accomplished through the use of multiple tools and methods,” and that “implementation of GCP principles should be a thoughtful, deliberative, and risk-based process as clinical trials can vary greatly and certain aspects of GCP may not be applicable to every trial” [19]. A draft version of the ICH E6(R3) principles is now available, which addresses the need for greater flexibility in the current research environment [20].

Our study has several strengths, including the composition of our sample, with participants who represented global professional clinical research networks and who were purposefully selected based on geographic diversity, professional role, and institutional type. Our work also includes the perspectives of commercial trialists, extending previously voiced critiques of ICH E6 GCP [3] by non-commercial trialists. In addition, diversity in the location of clinical trials is another strength, with participants representing clinical research efforts in 124 different countries.

Conversely, the geographic origin of our participant sample represents a limitation, as the bulk of IDI participants were from Europe and North America. Only a small percentage of IDI participants represented Asia and Latin America, and none were from African nations. However, a recent survey on the ICH E6 GCP revision among stakeholders in Japan [21] elicited similar findings to our interviews, including the need to clarify the scope of E6 GCP, modernize monitoring methods, and allow for flexibility in requirements for quality and procedures. This survey was based on the survey conducted by CTTI for the initial phase of this project [10]. An additional limitation of our study is that participants in the original CTTI survey who expressed interest in participating in the interview were quite invested in improvements to ICH E6 GCP. Therefore, a sample of participants less interested in improving ICH E6 GCP may have voiced different aspirations for amending the guidance.

CTTI provided the full report on the project's findings to ICH in March 2020 for their consideration. It is our hope that with revision to ICH E6 GCP actively underway, these findings will contribute to further refinement of the E6 GCP guidelines.

## Funding

The authors disclosed receipt of the following financial support for the research, authorship, and/or publication of this article: Funding for this work was made possible, in part, by the 10.13039/100000038U.S. Food and Drug Administration [cooperative agreement U18FD005292 and grant R18FD005292]. Views expressed in written materials or publications and by speakers and moderators do not necessarily reflect the official policies of the 10.13039/100000016Department of Health and Human Services, nor does any mention of trade names, commercial practices, or organization imply endorsement by the United States Government. Partial funding was also provided by pooled membership fees from the Clinical Trials Transformation Initiative's (CTTI) member organizations.

## Declaration of competing interest

The authors declare that they have no known competing financial interests or personal relationships that could have appeared to influence the work reported in this paper.

## Data Availability

The authors are unable to provide the data because it is qualitative and identifiable.
