# Supplementary material for: Stakeholders’ views on the most and least helpful aspects of the ICH E6 GCP guideline and their aspirations for the revision of ICH E6(R2)
Source: Contemp Clin Trials Commun. 2022 Aug 17;29:100983. doi: 10.1016/j.conctc.2022.100983 (PMC9468347; doi:10.1016/j.conctc.2022.100983)
Supplement: Multimedia component 1 [file mmc1.docx]

**Supplemental Appendix A. Geographic location of participants’ research^a^ (n=23).**

| **Region/ Country** | **n (%)** |
| --- | --- |
| **East Asia and Pacific** | **17 (73.9)** |
| Australia | 13 (56.5) |
| Cambodia | 2 (8.7) |
| China | 7 (30.4) |
| Indonesia | 5 (21.7) |
| Japan | 6 (26.1) |
| Malaysia | 6 (26.1) |
| New Zealand | 10 (43.5) |
| Philippines | 6 (26.1) |
| Singapore | 8 (34.8) |
| South Korea | 10 (43.5) |
| Taiwan | 8 (34.8) |
| Thailand | 6 (26.1) |
| Vietnam | 3 (13.0) |
| **Europe and Central Asia** | **21 (91.3)** |
| Albania | 1 (4.35) |
| Andorra | 1 (4.35) |
| Armenia | 1 (4.35) |
| Austria | 14 (60.9) |
| Azerbaijan | 1 (4.35) |
| Belarus | 5 (21.7) |
| Belgium | 19 (82.6) |
| Bosnia and Herzegovina | 2 (8.7) |
| Bulgaria | 7 (30.4) |
| Croatia | 7 (30.4) |
| Cyprus | 2 (8.7) |
| Czechia (Czech Republic) | 12 (52.2) |
| Denmark | 13 (56.5) |
| Estonia | 5 (21.7) |
| Finland | 13 (56.5) |
| France | 17 (73.9) |
| Georgia | 3 (13.0) |
| Germany | 20 (87.0) |
| Greece | 10 (43.5) |
| Greenland | 1 (4.35) |
| Hungary | 9 (39.13) |
| Iceland | 3 (13.0) |
| Ireland | 15 (65.2) |
| Italy | 18 (78.3) |
| Kosovo | 1 (4.35) |
| Latvia | 7 (30.4) |
| Liechtenstein | 1 (4.35) |
| Lithuania | 8 (34.8) |
| Luxembourg | 4 (17.4) |
| Malta | 2 (8.7) |
| Moldova | 4 (17.4) |
| Montenegro | 1 (4.35) |
| Netherlands | 16 (69.6) |
| North Macedonia (Formerly Macedonia) | 1 (4.35) |
| Norway | 13 (56.5) |
| Poland | 13 (56.5) |
| Portugal | 9 (39.13) |
| Romania | 8 (34.8) |
| Russia | 11 (47.8) |
| Serbia | 6 (26.1) |
| Slovakia | 8 (34.8) |
| Slovenia | 8 (34.8) |
| Spain | 17 (73.9) |
| Sweden | 14 (60.9) |
| Switzerland | 13 (56.5) |
| Turkey | 8 (34.8) |
| Ukraine | 8 (34.8) |
| United Kingdom | 20 (87.0) |
| **Latin America and Caribbean** | **13 (56.5)** |
| Argentina | 10 (43.5) |
| Brazil | 9 (39.13) |
| Chile | 8 (34.8) |
| Colombia | 6 (26.1) |
| Costa Rica | 2 (8.7) |
| Cuba | 1 (4.35) |
| Dominican Republic | 2 (8.7) |
| Ecuador | 3 (13.0) |
| El Salvador | 1 (4.35) |
| Guatemala | 3 (13.0) |
| Jamaica | 1 (4.35) |
| Mexico | 7 (30.4) |
| Panama | 3 (13.0) |
| Paraguay | 2 (8.7) |
| Peru | 5 (21.7) |
| Uruguay | 3 (13.0) |
| Venezuela | 5 (21.7) |
| **Middle East and North Africa** | **9 (39.13)** |
| Bahrain | 1 (4.35) |
| Egypt | 4 (17.4) |
| Iran | 1 (4.35) |
| Israel | 6 (26.1) |
| Jordan | 1 (4.35) |
| Kuwait | 1 (4.35) |
| Lebanon | 3 (13.0) |
| Oman | 1 (4.35) |
| Qatar | 1 (4.35) |
| Saudi Arabia | 2 (8.7) |
| Tunisia | 3 (13.0) |
| United Arab Emirates | 2 (8.7) |
| Yemen | 1 (4.35) |
| **North America** | **18 (78.3)** |
| Canada | 14 (60.9) |
| United States of America | 18 (78.3) |
| **South Asia** | **8 (34.8)** |
| Bangladesh | 3 (13.0) |
| India | 8 (34.8) |
| Nepal | 1 (4.35) |
| Pakistan | 1 (4.35) |
| Sri Lanka | 1 (4.35) |
| **Sub-Saharan Africa** | **13 (56.5)** |
| Benin | 2 (8.7) |
| Botswana | 1 (4.35) |
| Burkina Faso | 1 (4.35) |
| Cameroon | 1 (4.35) |
| Congo, Democratic Republic of the | 1 (4.35) |
| Cote d'Ivoire | 1 (4.35) |
| Ethiopia | 2 (8.7) |
| Gabon | 1 (4.35) |
| Gambia | 2 (8.7) |
| Ghana | 1 (4.35) |
| Guinea | 1 (4.35) |
| Kenya | 3 (13.0) |
| Malawi | 3 (13.0) |
| Mozambique | 3 (13.0) |
| Namibia | 1 (4.35) |
| Nigeria | 1 (4.35) |
| Rwanda | 3 (13.0) |
| Senegal | 1 (4.35) |
| Seychelles | 1 (4.35) |
| Sierra Leone | 2 (8.7) |
| South Africa | 11 (47.8) |
| Sudan | 1 (4.35) |
| Tanzania | 5 (21.7) |
| Uganda | 4 (17.4) |
| Zambia | 3 (13.0) |
| Zimbabwe | 3 (13.0) |

^a^ The regional headers represent the total number of participants and the percentage of the study population who conduct research in one of the countries in that region. The country subheaders represent the total number of participants and the percentage of the study population who conduct research in that country.
